# Selectivity and Sociality: Aggression and Affiliation Shape Vole Social Relationships

**DOI:** 10.3389/fnbeh.2022.826831

**Published:** 2022-03-07

**Authors:** Nicole S. Lee, Annaliese K. Beery

**Affiliations:** ^1^Department of Psychological and Brain Sciences, Colgate University, Hamilton, NY, United States; ^2^Department of Integrative Biology, University of California, Berkeley, Berkeley, CA, United States

**Keywords:** aggression, affiliation, prairie vole (*Microtus ochrogaster*), meadow vole (*Microtus pennsylvanicus*), selectivity, social motivation, tolerance

## Abstract

The formation of selective social relationships is not a requirement of group living; sociality can be supported by motivation for social interaction in the absence of preferences for specific individuals, and by tolerance in place of social motivation. For species that form selective social relationships, these can be maintained by preference for familiar partners, as well as by avoidance of or aggression toward individuals outside of the social bond. In this review, we explore the roles that aggression, motivation, and tolerance play in the maintenance of selective affiliation. We focus on prairie voles (*Microtus ochrogaster*) and meadow voles (*Microtus pennsylvanicus*) as rodent species that both exhibit the unusual tendency to form selective social relationships, but differ with regard to mating system. These species provide an opportunity to investigate the mechanisms that underlie social relationships, and to compare mechanisms supporting pair bonds with mates and same-sex peer relationships. We then relate this to the role of aggression in group composition in a comparative context.

## Introduction

Sociality (a.k.a. group living) takes many forms across social species, such that groups differ in size, composition, and the role of specific, selective relationships. Many attempts have been made to characterize types of social groups—for example, distinguishing between those that are large, transient, and gregarious vs. those that are smaller, more stable, and comprised of defined roles or relationships (Hinde, 1976; Lidicker and Patton, 1987; Lee, 1994; Lacey and Sherman, 2007; Clutton-Brock and Lukas, 2012; Hofmann et al., 2014; Lukas and Clutton-Brock, 2018; Kappeler, 2019; Bales et al., 2021). As would be expected from the diversity of group types, there are multiple neurobiological routes to supporting life in social groups. By taking advantage of natural variations in social behavior, we can hope to better understand both the unity and diversity in the biological underpinnings of different types of sociality. In this review we focus on lessons from selective social groups, and the interplay between affiliation and aggression in the maintenance of selectivity.

Only about 5–10% of rodent species are classified as group living (Lacey and Sherman, 2007; Lukas and Clutton-Brock, 2013). Among those, many species (including the most commonly used laboratory rodents) are gregariously social, forming loose aggregations with conspecifics without maintenance of repeated, targeted social interactions (Lee, 1994). Thus, only a small percentage of rodent species display the enduring and specific “selective” relationships that make it possible to study mechanisms underlying social bonding. Selective social preferences for known individuals are often assessed in laboratory settings using the partner preference test, a 3-h social choice test between an unfamiliar “stranger” and a familiar “partner” in a three-chambered apparatus (Williams et al., 1992; Beery, 2021). Selective preference for familiar individuals has been most commonly demonstrated in the genus *Microtus*, including socially monogamous prairie voles and mandarin voles, and seasonally social meadow voles (*opposite-sex*: Williams et al., 1992; Parker et al., 2001; Yu et al., 2013; Wu et al., 2018; *same-sex*: Parker and Lee, 2003; Beery et al., 2009; Beery and Shambaugh, 2021). Selectively social species such as voles spend significantly more time with partners than with strangers in these tests, whereas mice, rats, and degus do not prefer familiar individuals under typical conditions, and may prefer social novelty (Moy et al., 2004; Smith et al., 2015; Schweinfurth et al., 2017; Beery et al., 2018; Insel et al., 2020; Beery and Shambaugh, 2021; Hackenberg et al., 2021).

**FIGURE 1:**
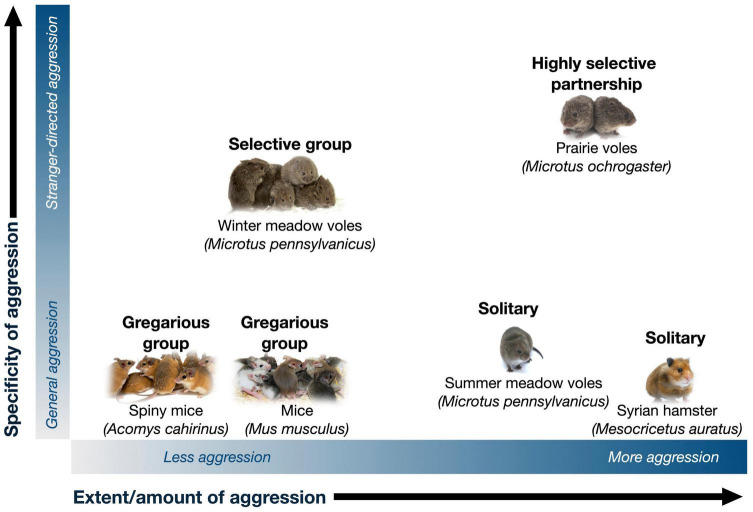
Together with affiliation, aggression shapes the presence and nature of social groups. The vast majority of rodents are solitary, such as the Syrian hamster. Differences in the amount or extent of aggression (*x*-axis) shape group formation, with low aggression and territoriality promoting gregarious group structures (e.g., mice, rats, and spiny mice) that may be flexible in size and composition. The generality vs. specificity (*y*-axis) of how aggression is targeted shapes the selectivity of groups for specific known members. Selectivity may be maintained by “prosocial” factors such as motivation for social bonds, as well as by lack of aggression and intolerance toward group members vs. strangers. Highly selective species such as prairie voles exhibit both high levels of affiliation and stranger aggression, while less selective groups such as winter-phenotype meadow voles show preference for familiar individuals in the absence of social motivation or intense aggression. Photo credits: *solitary rodent* (Syrian hamster; The Rohit CC-BY NC 2.0), *gregarious groups* (Mice; Pixabay user Kapa65; Spiny mice; Aubrey Kelly by permission), *selective groups* (meadow voles and prairie voles; Beery Lab).

Depending on context and social structure, aggression can serve to either promote or reduce social behavior (Figure 1). When social relationships are stable and selective, social groups may be closed or less flexible, and therefore aggression may serve to promote social behavior among members of a social group by excluding outsiders. On the other hand, lack of familiarity preference may be a key component of flexible and gregarious social groups, such as those formed by mice, rats, degus, and spiny mice (Beery et al., 2018; Insel et al., 2020; Kelly and Seifert, 2021; Lidhar et al., 2021). In these kinds of groups, aggression reduces social behavior; conversely, reduction in aggression promotes gregariousness. We focus on the role of selective aggression in affiliative social behavior in voles, then discuss how reduction in aggression and anxiety promote social behavior in gregarious animals more broadly.

## Specificity of Aggression Promotes Selective Groups

Selective preference for familiar individuals may result from aggression toward and avoidance of unfamiliar conspecifics, as well as potential prosocial motivation toward familiar individuals (Figure 1, selective groups). Selective social motivation vs. “tolerance” can be difficult to distinguish, and understanding their relative contributions has benefited from distinct assessment of social motivation, preference, and aggression.

### Highly Selective Partnerships: Affiliation and Aggression in Monogamous Prairie Voles

Prairie voles are socially monogamous rodents that have been well-studied for their pair bonding behavior and biparental care (reviewed in Carter, 2017; Gobrogge et al., 2017; Walum and Young, 2018; Kenkel et al., 2021). Prairie voles are native to grasslands, primarily in the midwestern United States, where repeated live trapping has revealed that social groups are often comprised of male-female reproductive pairs, characterized by overlapping home ranges, shared nests, territoriality, and mate-guarding (Getz et al., 1993; Getz and Carter, 1996; Madrid et al., 2020). In addition to mate partnerships, prairie voles can also form non-reproductive relationships with same-sex conspecifics or “peers” (DeVries et al., 1997; Beery et al., 2018; Lee et al., 2019); these female-female relationships are selective and enduring, much like pair bonds with mates. Laboratory studies indicate that relationships in prairie voles are maintained by stranger aggression and reinforced by social reward, leading to high levels of selectivity (Figure 1, highly selective partnership).

Selective aggression occurs in males and females, in the field and lab, and is directed at same- and opposite-sex conspecifics (reviewed in Young et al., 2011). It may promote mate-guarding, territory defense, and more generally maintain the pair bond. Experiments in male prairie voles have elucidated the timing and target-specificity of this response. Aggression is elevated following cohabitation with a mate; males housed with a female for 24 h exhibited significantly more stranger-directed aggression in a resident-intruder test than unmated males (Winslow et al., 1993; Insel et al., 1995; Wang et al., 1997). Although mated males exhibited aggression toward strangers of both sexes, female-directed aggression after a 24-h cohousing interval was less intense than male-directed aggression (Wang et al., 1997). However, after 2 weeks of cohabitation with a mate, males displayed intense aggression toward even female strangers (Aragona et al., 2006).

Maternal/pregnant females and females paired with males (with or without mating for different intervals) also displayed increased aggression toward female strangers compared to females housed with females (Bowler et al., 2002). This aggression peaked after 12 days of opposite-sex cohabitation. In trios consisting of two sibling or non-sibling females and an unrelated male housed together for 3 days (both sexes sexually experienced), non-sibling female groups exhibited significantly more female-female aggression than sibling female groups (Firestone et al., 1991). This suggests that the presence of a male reduces female tolerance for unfamiliar females.

The mechanisms underlying both affiliation and aggression in prairie vole social relationships have been extensively explored (Table 1), with many signaling pathways involved in both processes. For example, the neuropeptides oxytocin and vasopressin are both implicated in social bond formation in prairie voles, as well as in aggression. Oxytocin has a long-appreciated role in pair bond formation in females (Williams et al., 1992; Insel and Hulihan, 1995) and more recently in males (Liu et al., 2001; Ophir et al., 2012; Duclot et al., 2016; Johnson et al., 2016). There is growing appreciation that oxytocin mediates both prosocial and antisocial behaviors, and there is evidence for overlapping neural circuitry mediating these seemingly opposing but similarly oxytocin-dependent processes (Bartz et al., 2010; Chen et al., 2011; van Anders et al., 2013; Beery, 2015; Anacker et al., 2016a; de Jong and Neumann, 2018; Steinman et al., 2019). Several studies highlight a role of oxytocin in aggressive behavior in prairie voles. Female prairie voles treated with oxytocin shortly after birth exhibited more aggression toward a same-sex stranger in a neutral arena after brief exposure to a male, compared to control or oxytocin antagonist-treated females (Bales and Carter, 2003). Oxytocin receptor density in the BNST of female prairie voles was associated with increased aggression toward unfamiliar voles (Beery et al., 2021), and in males, an *Oxtr* genotype that is associated with striatal oxytocin receptor expression and bond formation (King et al., 2016; Ahern et al., 2021) is also strongly associated with aggression toward strangers (Vahaba et al., 2021). Oxytocin also mediates aggression and social anxiety in many other rodent species, including rats, mice, naked mole rats, California mice, hamsters, and Mongolian gerbils (reviewed in Campbell, 2008; de Jong and Neumann, 2018; Steinman et al., 2019).

**TABLE 1:** A summary of the signaling pathways involved in prairie vole affiliation and aggression.

*Promote/positively associated with*....	Affiliation		Aggression	
	♂	♀	♂	♀
Oxytocin signaling pathway	✓ (OTR)	✓ (OTR)	✓ (OTR)	✓ (OTR)
Vasopressin signaling pathway	✓ (V1aR)	?	✓ (V1aR)	?
Dopamine signaling pathway	✓ (D2)	✓ (D2)	✓ (D1)	✓ (D1)
Opioid signaling pathway	✓ (μ)	✓ (μ)	✓ (κ)	✓ (κ)
Corticosterone	✓	X	X	?

*Check marks denote pathways that promote or are positively associated with affiliation or aggression. Parentheses indicate implicated receptor subtypes. X’s indicate pathways that do not promote affiliation or aggression, either by having no effect or by instead reducing the behavior. Question marks denote pathways that have not yet been investigated.*

The role of vasopressin in aggressive behavior was one of its earliest known social functions in the brains of mammals (Albers, 2012). In male prairie voles, activation of V1aR receptors in the ventral pallidum is necessary for pair bond formation (Lim and Young, 2004). Pair bonding also leads to an increase of vasopressin in the anterior hypothalamus of male prairie voles, where activation of V1a receptors promotes aggressive behavior, while blockade of V1a receptors decreases aggressive behavior (Gobrogge et al., 2009). Across species, the role of vasopressin in aggression is a topic of extensive study.

Selective post-mating aggression in prairie voles is also mediated by dopaminergic and opioid signaling pathways. Pair bond maintenance was associated with upregulation of dopamine D1 type receptors in the nucleus accumbens in male prairie voles (Aragona et al., 2006), and with upregulated mRNA expression of genes encoding D1-type receptors in males and females (Resendez et al., 2016). Peripheral blockade of κ-opioid receptors, as well as blockade in the nucleus accumbens shell, abolished selective aggression in prairie vole males and females (Resendez et al., 2012). Furthermore, κ-opioid receptors and D1 receptors interacted to maintain pair bonds (Resendez et al., 2016). Dopamine and vasopressin in the nucleus accumbens, anterior hypothalamus, and central amygdala have also been found to be involved in maintenance-related aggression in male prairie voles (reviewed in Gobrogge and Wang, 2011).

Corticosteroids also play an important role in prairie vole social behavior. For example, acute corticosterone administration has a sexually dimorphic effect on prairie vole pair bonding, facilitating the formation of partner preferences by males for females (without inducing male territorial aggression), but inhibiting bonding of females to males (DeVries et al., 1996; Blondel and Phelps, 2016). While the involvement of gonadal steroid hormones in aggression is well established in multiple species (reviewed in Soma et al., 2008), there is some evidence suggesting that they are less important in prairie vole aggression as well as affiliation (reviewed in Carter and Perkeybile, 2018). For example, castration failed to inhibit aggression or pair bonding in adult male prairie voles (Demas et al., 1999; Carter and Perkeybile, 2018). However, neonatal castration did successfully disrupt pair bonding (Cushing et al., 2003).

Although aggression mediates selectivity in prairie vole mate and peer relationships, the relative importance of prosocial motivation varies across relationship type. Prairie vole mate relationships rely on dopamine and opioid signaling (Aragona et al., 2003, 2006; Resendez et al., 2012, 2016), and are associated with behavioral reward in socially conditioned place preference (sCPP) tests and operant studies using a social reward (Ulloa et al., 2018; Goodwin et al., 2019; Beery et al., 2021). While dopamine signaling is not essential for the formation of peer relationships in prairie voles (Lee and Beery, 2021), female prairie voles find familiar peers more motivating than strangers or an empty chamber, and condition toward socially associated cues in the sCPP test (Beery et al., 2021; Lee and Beery, 2021). Because prairie voles direct their affiliation and aggression at specific targets, both aspects of behavior likely contribute to their highly selective social groups. Increased affiliation toward members of an ingroup and decreased affiliation toward outgroup individuals has also been demonstrated in humans (e.g., Faulkner et al., 2004; Navarrete and Fessler, 2006; Dreu et al., 2010, 2011; Oaten et al., 2011; Hruschka and Henrich, 2013; Neuberg and Schaller, 2016) and other species.

### Selective Groups: Selectivity and Flexibility in Meadow Vole Social Relationships

Meadow voles are not socially monogamous, and because they are closely related to, yet behaviorally distinct from prairie voles, they provide an opportunity to assess similarities and differences in behavior and mechanism across species and mating systems (e.g., Wang et al., 1994; Kingsbury et al., 2012; Inoue et al., 2013; Lee et al., 2019; Beery et al., 2021). Meadow voles also provide a within-species opportunity to examine mechanisms underlying variation in social grouping, as they exhibit seasonal shifts in social behavior. Meadow voles live in grassy fields, woodlands, and marshes throughout most of Canada and northern and eastern parts of the United States. Wild meadow voles are intolerant of conspecifics during the reproductive season: In the summer (long day lengths, LD), females in particular form exclusive territories and (except for mating) are aggressive toward adult voles of both sexes. However, in the winter (short day lengths, SD), meadow voles form tolerant, mixed-sex communal huddling groups, have overlapping home ranges, and share nests with conspecifics (Madison, 1980; McShea and Madison, 1984; Ferkin and Seamon, 1987; Madison and McShea, 1987). These groups typically begin with undispersed family members joined by immigrant males (Madison et al., 1984), but by early January, predation and acceptance of new group members lead to groups that are no longer kin-based. In the Spring, groups close to new members (Madison and McShea, 1987). Meadow vole winter social groups are thus selective, but somewhat flexible (Figure 1).

Seasonal changes in social organization in wild meadow voles are mirrored in the laboratory under changing photoperiods: short day lengths promote greater social huddling and reduced anxiety behavior (Parker and Lee, 2003; Ossenkopp et al., 2005; Beery et al., 2008, 2009; Lee et al., 2019). Because of this daylength-dependent variation in social group formation, meadow voles have been used to study the environmental and neural factors that alter the propensity to form selective same-sex relationships (Beery and Zucker, 2010; Beery et al., 2014; Ondrasek et al., 2015; Anacker et al., 2016a; Beery, 2019).

Meadow voles exhibit selective preferences for familiar same-sex and opposite-sex individuals in partner preference tests (Parker and Lee, 2003; Beery et al., 2009; Stetzik et al., 2018; Beery and Shambaugh, 2021), and can simultaneously form such preferences for multiple familiar individuals (Beery et al., 2009). Interestingly, relative preference for the partner vs. the stranger is sometimes found in both SD- and LD-housed females (Ondrasek et al., 2015; Goodwin et al., 2019), although SD-housed females may huddle more than their LD-housed counterparts with both partners and strangers (Beery et al., 2008). Increased social huddling, including tolerance of strangers, is an important factor in winter group formation, as meadow vole groups accommodate immigration throughout the winter before becoming fixed in early spring (Madison and McShea, 1987). Meadow voles thus exhibit both selective, familiarity-based affiliation, and increased social flexibility and gregariousness during winter months.

While meadow voles naturally cohabit in winter social groups, social reward does not appear to play an important role in group formation. Meadow voles housed in SDs in the lab show no sign of social reinforcement in either sCPP tests (Goodwin et al., 2019) or in operant conditioning tests with social rewards (Beery et al., 2021). Partner preferences for same-sex peers are also dopamine-independent in this species (Beery and Zucker, 2010). Thus, social motivation does not seem to explain winter grouping behavior.

Social tolerance—mediated *via* seasonal reduction in anxiety and aggression—provides a better explanation for winter sociality, and seasonal changes in tolerance and aggression have been documented in multiple field studies (Madison, 1980; McShea and Madison, 1984; Ferkin and Seamon, 1987; Madison and McShea, 1987; McShea, 1990). Laboratory studies provide several lines of support for this hypothesis. Behaviorally, meadow voles housed in short day lengths exhibit greater social interaction with strangers and less anxiety-like behavior (Ossenkopp et al., 2005; Lee et al., 2019), while exogenous stressors that elevate corticosterone signaling impair the formation of new non-reproductive relationships (Anacker et al., 2016b). Anxiety may be modulated by photoperiod-mediated changes in HPA axis activity: glucocorticoid metabolites are lower in SD-housed voles and neural receptor densities of corticotropin releasing factor receptors (CRF1 and CRF2) change with day length in opposing directions, consistent with seasonal decline in anxiety (Beery et al., 2014; Anacker et al., 2016b). Decreased anxiety and increased investigation likely support the formation of groups, as well as the continued slow acceptance of new members throughout early winter.

### Interspecific Comparisons

The relative importance of factors promoting vs. opposing social interaction differs across vole species, with both selective aggression and social motivation playing a larger role in prairie voles compared to meadow voles. While both prairie and meadow voles form partner preferences for same-sex peers, studies comparing peer relationships in these species indicate that prairie voles spend more time in contact with a partner than do meadow voles (Beery and Shambaugh, 2021). As described above, social reward plays a role in mate and peer relationships in prairie but not meadow voles, and social reward may play a larger role in mate vs. peer relationships in the former species (Goodwin et al., 2019; Beery et al., 2021; Lee and Beery, 2021). Specific affiliation plays an important role in determining who is in a group.

Just as factors *promoting* social interaction differ between species, so do factors *preventing* interaction. Selective aggression toward unfamiliar same-sex conspecifics was higher in social interaction tests in prairie voles than in meadow voles (Lee et al., 2019), and female prairie voles exhibited significantly more stranger-directed aggression than meadow voles in operant conditioning trials when they gained access to the stranger chamber (Beery et al., 2021). Like prairie vole mate relationships, peer relationships in this species are maintained in part by aggression toward unfamiliar individuals. In contrast, social tolerance is an important feature of meadow vole peer affiliation, demonstrated by low aggression toward unfamiliar conspecifics, and suggested by field data on winter tolerance. This low aggression may be particularly important in shaping group size, or how closed groups are to new members. Together, affiliation and aggression may substantially shape social groups, thereby contributing to potential interspecific differences in the nature of these groups.

## Reduction of Aggression and Anxiety Promotes Gregarious Interactions

For gregarious groups, familiarity does not play a central role in group formation. Instead, gregarious social groups may result from a generalized lack of aggression, together with prosocial factors (e.g., motivation) that more actively promote grouping (Figure 1, gregarious groups). In many species, there is an inverse relationship between aggression and social behavior—that is, aggression and social anxiety drive social avoidance, and a tendency to be solitary, while the absence of aggression in combination with other factors may lead to the formation of social groups. Opposing roles of affiliation and aggression/anxiety have been documented in rodents and birds (detailed below), among many other taxonomic groups.

In rodents, the relationship between anxiety and social interaction is so well-defined that the primary use of the social interaction test is as an indicator of anxiety, with low social interaction (e.g., sniffing or grooming) associated with high anxiety (reviewed in File and Seth, 2003). This association also forms the basis for experimental manipulations investigating anxiogenic and anxiolytic effects of specific factors of interest, especially in conjunction with other common tests of anxiety-like behavior in rodents. For example, in rats, greater social contact in the social interaction test is often correlated with lower anxiety in measures such as the elevated plus maze and light-dark box (Starr-Phillips and Beery, 2014; Sparling et al., 2018). Rats that exhibited higher anxiety-like behavior were also slower to learn helping behavior in the form of freeing a trapped conspecific from a restrainer (Ben-Ami Bartal et al., 2014).

Aggression is also associated with reduced social behavior. Studies in rats have shown that central oxytocin reduces aggression and increases affiliative behavior, although endogenous oxytocin signaling also plays a critical role in promoting aggression (e.g., Calcagnoli et al., 2015; Oliveira et al., 2021). Aggression has also been hypothesized to shape group size in birds, many of which exhibit seasonal differences in social behavior, from exclusive territories in the breeding season to flocks of several to thousands of individuals in the non-breeding season (Goodson and Kingsbury, 2011; Goodson et al., 2012a,b). There is evidence that oxytocin (Goodson et al., 2009) and vasopressin (Kelly et al., 2011) support larger flock sizes in gregarious zebra finches. Furthermore, in comparisons across territorial and gregarious (i.e., flocking) finch species, nonapeptides have been found to mediate flocking behavior. For example, differences in oxytocin receptor binding density in the lateral septum were found between territorial and gregarious finches (Goodson et al., 2009). Thus, while amount/extent of aggression may determine group sizes—as in seasonally flocking finches—specificity of aggression—as in voles—shapes group membership.

## Conclusion

The role of aggression in social behavior is dependent on species and social context. Aggression can reduce social interactions by promoting avoidance and solitary living. Conversely, reduction in aggression may support group living in the form of gregarious and flexible social groups. Aggression also plays an essential role in promoting selective social behavior—at topic that has received less attention. In prairie voles, aggression toward strangers enhances selectivity toward partners. The winter transition to sociality in species such as meadow voles may rely on relaxation of selective aggression as well as reduction in anxiety to promote larger and more flexible groups.

## Author Contributions

Both authors conceived, wrote, and edited this mini-review.

## Conflict of Interest

The authors declare that the research was conducted in the absence of any commercial or financial relationships that could be construed as a potential conflict of interest.

## Publisher’s Note

All claims expressed in this article are solely those of the authors and do not necessarily represent those of their affiliated organizations, or those of the publisher, the editors and the reviewers. Any product that may be evaluated in this article, or claim that may be made by its manufacturer, is not guaranteed or endorsed by the publisher.
